# Comparing a pre-defined versus deep learning approach for extracting brain atrophy patterns to predict cognitive decline due to Alzheimer’s disease in patients with mild cognitive symptoms

**DOI:** 10.21203/rs.3.rs-3569391/v1

**Published:** 2023-11-08

**Authors:** Ida Arvidsson, Olof Strandberg, Sebastian Palmqvist, Erik Stomrud, Nicholas Cullen, Shorena Janelidze, Pontus Tideman, Anders Heyden, Karl Åström, Oskar Hansson, Niklas Mattsson-Carlgren

**Affiliations:** Lund University; Lund University; Lund University; Lund University; Lund University; Lund University; Lund University; Lund University; Lund University; Lund University; Lund University

**Keywords:** Alzheimer’s disease, cognitive decline, deep learning

## Abstract

**Background:**

Predicting future Alzheimer’s disease (AD)-related cognitive decline among individuals with subjective cognitive decline (SCD) or mild cognitive impairment (MCI) is an important task for healthcare. Structural brain imaging as measured by magnetic resonance imaging (MRI) could potentially contribute when making such predictions. It is unclear if the predictive performance of MRI can be improved using entire brain images in deep learning (DL) models compared to using pre-defined brain regions.

**Methods:**

A cohort of 332 individuals with SCD/MCI were included from the Swedish BioFINDER-1 study. The goal was to predict longitudinal SCD/MCI-to-AD dementia progression and change in Mini-Mental State Examination (MMSE) over four years. Four models were evaluated using different predictors: 1) clinical data only, including demographics, cognitive tests and *APOE* e4 status, 2) clinical data plus hippocampal volume, 3) clinical data plus all regional MRI gray matter volumes (N=68) extracted using FreeSurfer software, 4) a DL model trained using multi-task learning with MRI images, Jacobian determinant images and baseline cognition as input. Models were developed on 80% of subjects (N=267) and tested on the remaining 20% (N=65). Mann-Whitney U-test was used to determine statistically significant differences in performance, with p-values less than 0.05 considered significant.

**Results:**

In the test set, 21 patients (32.3%) progressed to AD dementia. The performance of the clinical data model for prediction of progression to AD dementia was area under the curve (AUC)=0.87 and four-year cognitive decline was R^2^=0.17. The performance was significantly improved for both outcomes when adding hippocampal volume (AUC=0.91, R^2^=0.26, p-values <0.05) or FreeSurfer brain regions (AUC=0.90, R^2^=0.27, p-values <0.05). Conversely, the DL model did not show any significant difference from the clinical data model (AUC=0.86, R^2^=0.13). A sensitivity analysis showed that the Jacobian determinant image was more informative than the MRI image, but that performance was maximized when both were included.

**Conclusions:**

The DL model did not significantly improve the prediction of clinical disease progression in AD, compared to regression models with a single pre-defined brain region.

## Introduction

Alzheimer’s disease (AD) affects millions of individuals worldwide. AD dementia is characterized by a prolonged prodromal phase in which amyloid pathology accumulates in the brain before cognitive decline starts (1). The subsequent onset of tau pathology and atrophy tracks more closely with occurrence of symptoms in the early phase of the disease in which individuals experience only subjective cognitive decline (SCD), or cognitive decline that qualify for mild cognitive impairment (MCI) (1, 2). However, despite this understanding of the pathophysiological cascade of AD, it is not straight forward which individuals, who are present in a clinical setting with SCD/MCI, will progress to AD dementia (early AD) versus those that remain with stable SCD/MCI or develop other dementias (non-AD) (3–5). It is also often unclear at which rate individuals with SCD/MCI will continue to decline cognitively, particularly due to varying biological resiliency to AD pathology at the individual level (6). With the recent breakthroughs in disease-modifying treatments (DMT) against AD (7, 8), it may be possible to alter the course of disease in patients with SCD/MCI due to AD. Given the heterogeneity of the SCD/MCI population, it is now urgent to bring forward methods that can guide physicians when making decisions about which patients that are most likely to benefit from receiving DMTs targeting AD pathology.

To improve the prognosis of AD-related cognitive changes, artificial intelligence (AI) could be useful. Features from clinical data and different biomarker modalities can automatically be extracted and combined, to guide in the discrimination between individuals who will remain with SCD/MCI and those who will be diagnosed with AD dementia. One of the most common imaging modalities used for this task is magnetic resonance imaging (MRI). Several previous studies have utilized AI and MRI for this, including for example (9–18). As can be seen in the review by Grueso et al. (19), the AI methods used vary but often include the support vector machine. However, in more recent studies the usage of deep learning (DL) and convolutional neural networks (CNN) have become frequent thanks to larger datasets and increased computational power. With DL it is possible to extract complex features from a large amount of data, such as a 3D MRI image. A review of publications regarding AD dementia detection using DL is given in (20), showing that MRI is the most widely available and used biomarker for this task, but a variety of DL models are used, including both voxel-based, slice-based, patch-based and region of interest-based. Despite this development in methods, there is a lack of studies that objectively compare the performance of AI methods with more intuitive methods, such as logistic or linear regression models with a restricted number of predictors of cognitive decline in the early stages of AD. In SCD/MCI patients, there is also a lack of studies for prediction of longitudinal cognitive decline using commonly used continuous measures of cognition, rather than progression to AD dementia as an outcome.

The main goal of the present study was to perform an unbiased comparison between models utilizing different baseline information for prediction of future cognitive decline in SCD/MCI patients. This is done to understand which variables hold the most information and which method is the most accurate. Two different predictions were done – progression from SCD/MCI to AD dementia within four years (binary outcome) and four-years Mini Mental State Examination (MMSE) slope (continuous outcome). The variables evaluated were demographic information, baseline cognition based on cognitive tests, *APOE* genotype, predefined volumetric variables, and MRI images. The models used were logistic and linear regression, random forest as well as a DL model consisting of a three-dimensional (3D) CNN. Understanding and evaluating the clinical usefulness of these models have rarely been done in an independent and prospective manner. The present study aims to answer whether most of the prognostic information about cognitive decline in an MRI scan is contained in features of the brain 1) which can be obtained through volumetric analysis of pre-specified brain regions, or 2) which can only be obtained through an AI model identifying novel and previously unspecified patterns of brain structure. In this sense, the present study can provide insight as to which level of abstraction prognostic information for AD dementia progression is found.

## Methods

### Cohort description

Individuals with SCD or MCI were included from the Swedish BioFINDER-1 study (clinical trial no. NCT01208675). The study protocol is described in detail before (21). Briefly, the consecutively recruited participants in BioFINDER-1 are aged between 60 and 80 years, perform ≥ 24 points on the MMSE, and have been referred to any of the participating memory clinics due to cognitive complaints. A neuropsychological assessment including a comprehensive test battery was used to classify participants as SCD or MCI as previously described (22). All patients with MCI were classified based on the DSM-5 criteria for MCI (23). Note however, that for this study the SCD and MCI groups were analyzed together, since the aim of the project was to develop methods that would be useful for longitudinal predictions in an unselected group of patients with cognitive complaints, prior to dementia. Exclusion criteria were cognitive impairment that could be better accounted for by another non-neurodegenerative condition, severe somatic disease, and current alcohol or substance abuse. Only patients with available baseline MRI scans and longitudinal cognitive follow-up of at least four years from baseline were included here. Demographic information (age, sex, education), *APOE* ε4 carriership status (negative/positive) and baseline cognition (MMSE score and Alzheimer’s Disease Assessment Scale [ADAS] delayed word recall) were collected for all individuals.

A random subset of 20% were used as a test set and was thus not in any way used during the model development phase. The remaining 80% were used as development set. To reduce the risk of overfitting and get an estimate of the uncertainty of the result, 10-fold cross-validation was used for the development set. The division into test and development sets was done such that there was no significant difference between train and test sets in the distribution of diagnosis, age, education, sex, or *APOE* status. The cross-validation folds were drawn such that the same ratio of early AD and non-AD was obtained for each fold, but otherwise randomly. An overview of the demographics for the test and development sets can be seen in Table 1.

### Study outcomes

There were two outcomes of interest. The primary binary outcome was four-year progression to AD dementia, where a clinical diagnosis of AD dementia during the four years follow-up was considered as a progression. Clinical status of AD dementia was evaluated according to the DSM-5 criteria for major neurocognitive disorders and recorded at each visit by a senior neuropsychologist and experienced memory disorder specialist. Additionally, a diagnosis of AD dementia was only used if the participant had an abnormal CSF profile consistent with AD pathological change. The diagnostic process is described in detail in (21). The primary continuous outcome was four-year cognitive decline as measured by the change from baseline in the MMSE score. MMSE is a measure of global cognition, and ranges from 0 to 30 where lower scores indicate worse cognition. Four-year cognitive decline was measured by fitting linear regression models for each individual separately using all available follow-up data within four years from baseline, then extracting the estimated regression slope.

### MRI procedures

T1-weighted MRI was performed on a 3T Skyra MRI scanner (Siemens Healthineers, Erlangen, Germany) producing a high-resolution anatomical MP-RAGE image (TR = 1950 ms, TE = 3.4 ms, 1 mm isotropic voxels, 178 slices). The MRI images were minimally processed using skull stripping, bias correction, and normalization to MNI152 template space (24) using ANTS (25). Cortical reconstruction and volumetric segmentation were performed with the FreeSurfer image analysis pipeline, as described previously (26). The Jacobian determinant (JD) images where computed based on the anatomical MRIs non-linear warp to template space and quantify the local deformations, wherein reduced brain matter and atrophy are gauged.

### Sets of predictors

**Figure 1:**
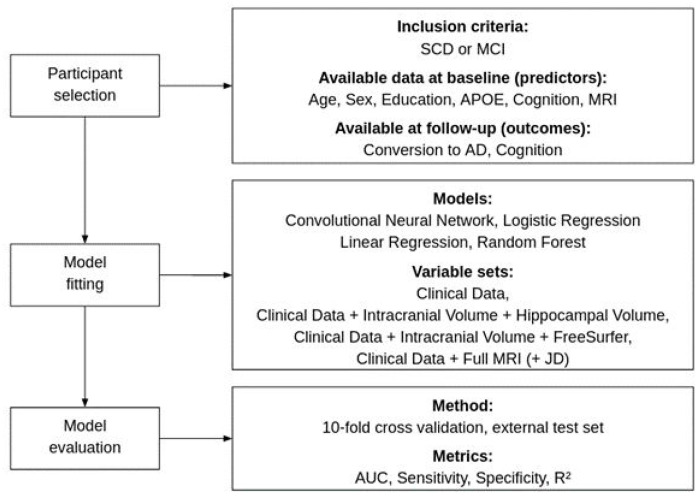
Overview of study.

Several *a priori* measurements are related to change in cognition, wherefor we investigated several different types of data and models, see Fig. 1. The first model [“Clinical data model”] utilized readily available demographics information (age, sex, and education), MMSE score, ADAS delayed word recall (27) and *APOE* status. The second model [“Hippocampal volume model”], used hippocampal volume (average of left and right hemisphere) as well as intracranial volume added to the clinical data model. The third model [“FreeSurfer model”], used regional brain volumes from the FreeSurfer pipeline together with intracranial volume added to the clinical data model. The fourth model [“DL model”] used whole brain MRI and JD images along with the clinical data variables in a CNN model. The different models are described in more detail below.

The study consisted of three parts: participant selection, model fitting, and model evaluation.

### Basic and Volumetric models

For the clinical data model and the hippocampal volume model we trained logistic regression models for prediction of progression to AD dementia and linear regression models for prediction of longitudinal cognitive decline. For the FreeSurfer models, random forest was used.

For all of the models the features were standardized by removing the mean and scaling to unit variance. The models were optimized using the Scikit-learn library (v. 0.22) in Python (v. 3.5) (28). For the random forest models, the random forest classifier was used for prediction of progression to AD dementia and the random forest regressor was used for prediction of longitudinal cognitive decline, both with default parameters.

### Deep learning models

CNNs work by learning a successively more complex representation of images across its increasing layers, where the earliest layers closest to the input image are activated by simple shapes such as edges, followed by more complex structures. This method of creating an increasing complex visual representation is similar to how the brain’s visual cortex processes images. We used the CNN architecture suggested but Spasov et al. (14), which is a parameter-efficient network, reducing the risk of overfitting when using small datasets, and has previously been proven successful (13, 14). We modified the network slightly for our settings, see Supplementary Fig. 1. The model utilizes both the MRI image, the JD image and the clinical data. The main modification done was to train the network for new tasks using multi-task learning (29), which reduces the risk of overfitting the model.

When using multi-task learning the network is trained for several tasks simultaneously, which for example can be beneficial when the dataset used is limited in size and thus the risk of overtraining is high. The multi-task learning was implemented by using three output layers, one for each of the tasks i) discrimination for four-year progression to AD with sigmoid activation and class weighted categorical cross-entropy loss ***L***_1_, ii) prediction of four-year cognitive decline measured with MMSE slope with linear activation and mean-squared-error loss ***L***_2_, and iii) prediction of hippocampal volume with linear activation and mean-squared-error loss ***L***_3_. Thus, each training example was used for all three tasks and the total loss function ***L*** was a weighted sum of the three individual ones with weights *w*_1_, *w*_2_, *w*_3_,

$$L=w_{1}L_{1}+w_{2}L_{2}+w_{3}L_{3}$$

1.

Depending on which task was the main one, the weighting of the different individual losses was modified. For the model discriminating four-year progression to AD, we used *w*_1_ = **1** and *w*_2_ = *w*_3_ = **0.025** which was found being a good configuration by testing values in the range 0-0.1, see Supplementary Table 1. Similarly, we used *w*_2_ = **1** and *w*_2_ = **1** and *w*_1_ = 0.025 for the model predicting MMSE slope, see Supplementary Table 2.

The size of our MRI images and JDs differed slightly from the sizes used in the work by Spasov et al. (14). To be able to use the same network architecture, the MRIs were cropped, and the JDs were padded with zeros. The MRIs were normalized to have voxel values in range [−1, 1] by subtracting the smallest voxel value in the entire MRI set, dividing by 0.5 times the largest voxel value in the entire MRI set and subtracting 1. No normalization of the JD images was done. The clinical data was all individually normalized to have values in range [0, 1], by removing the minimum value and dividing by the difference between the maximum and minimum value. The normalization technique was modified compared to the one used in (14), since better validation performance was obtained.

Similar to the settings used in (14), the network was trained for 50 epochs using the Adam optimizer with the same learning rate scheduler. The model was implemented based on the code provided by (14) but with the final layers modified to be able to use multi-task learning. The implementation was done in Python (version 3.8) using the Keras library (30) with TensorFlow (31) as backend and trained on a Nvidia Tesla V100 graphics card with 32GB VRAM.

### Statistical analysis

The primary analysis involved the models described above. A sensitivity analysis was also performed looking at the effect of including the MRI image only, the JD image only, or both in the DL model.

To improve model interpretability, canonical patterns of brain atrophy for the DL model were identified. Brain atrophy patterns are individualized in nature, so the block occlusion method was followed whereby parts of the image were systematically set to 0 and model performance was evaluated with these images and compared to images without any occlusion. Performing this procedure by systematically blacking out all parts of the image at different trials results in a whole brain atrophy pattern where it is assumed that regions whose blacking out results in a large decrease in model performance must be important to the DL model (32, 33). This was done for five non-AD and five early AD individuals. The average of the results from the ten folds and ten individuals were used for the final illustrations.

The performance metric of interest for the binary outcome of four-year progression to AD dementia was area under the curve (AUC). For the continuous outcome of four-year cognitive decline, the performance metric of interest was R^2^.

The model fitting procedure involved first performing 10-fold cross validation on the development set, where the training set was the part of the data used to determine model parameters and the validation set was the part of the development set which was held out during cross validation to evaluate model parameters without looking at the test set. Once model parameters were determined from this internal cross validation procedure, performance was finally evaluated on the previously unseen test set and reported as the mean for the ten folds.

Statistically significant differences in demographics were determined using p-values from Fisher’s exact test (sex) or t-test for independent samples (remaining variables). To determine statistically significant differences in performance on the test set for the different models, the results from the 10 folds were used in a Mann-Whitney U-test using the Scipy library (v. 1.4.1) in Python (v. 3.5). All p-values less than 0.05 were considered significant. Bootstrapping on the test set was used to estimate 95% confidence intervals (CI).

## Results

### Cohort characteristics

A total of 332 participants from the Swedish BioFINDER-1 study were included in the present analysis, whereof 223 participants were non-AD participants who were cognitive stable with SCD or MCI for at least four years and the remaining 109 participants were early AD participants with SCD or MCI at baseline who subsequently progressed to AD dementia. Before any analysis, 267 participants (80%) were assigned to a development cohort and 65 participants (20%) were assigned to the test cohort.

In the development cohort, the non-AD participants did not differ from early AD participants significantly on education (12.0 years vs 11.8 years average, p-value 0.69), sex (45.8% female vs 48.9% female, p-value 0.70) or intracranial volume (1139 cm^3^ vs 1116 cm^3^ average, p-value 0.39), but the early AD group had a higher ratio of MCI (69% MCI vs 42% MCI, p-value < 0.05), was older (71.8 years vs 70.2 years average, p-value < 0.05), had lower baseline MMSE (27.2 vs 28.2 average, p-value < 0.001), higher *APOE* ε4 allele presence (1.0 vs 0.4, p-value < 0.001), worse ADAS score (6.5 vs 4.1 average, p-value < 0.001) and smaller hippocampal volume (2972.6 mm^3^ vs 3327.1 mm^3^ average, p-value < 0.001). Most of these trends were also seen in test cohort, except that there were no significant differences in age (early AD 73.2 years vs non-AD 70.5% years, p-value 0.10).

### Performance for predicting four-year progression to AD

**Figure 2:**
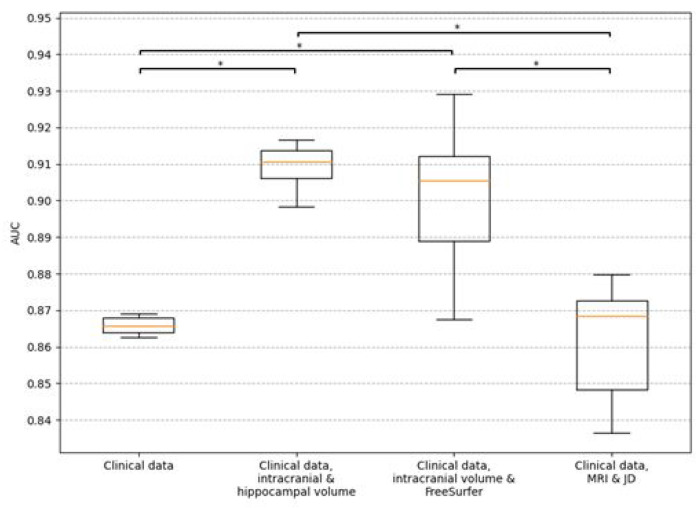
Box plots for AUC when predicting progression into AD dementia within four years. Results are for the test set using the different models from ten-fold cross-validation. The clinical data includes demographics (age, sex, and education), baseline cognition (MMSE score, ADAS delayed word recall) and APOE genotype. The orange lines show the median, the boxes span from the first quartile to the third quartile, and the whiskers extend from the box by 1.5 times the interquartile range. Significant differences (p-value < 0.05) are indicated with *.

**Figure 3:**
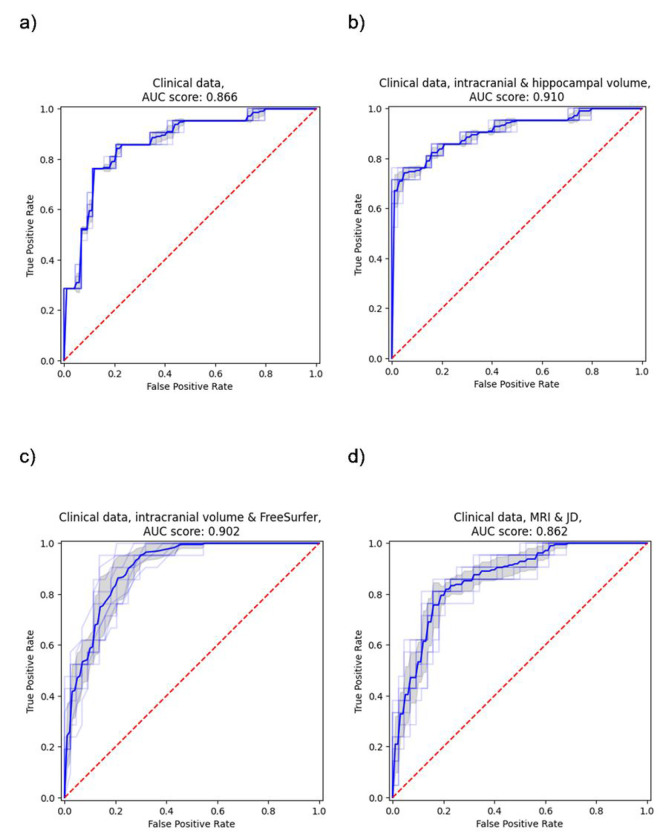
ROC curves and AUC for progression to AD dementia. Models using a) clinical data, b) clinical data, intracranial volume and hippocampal volume, c) clinical data, intracranial volume and FreeSurfer, d) clinical data, MRI and JD.

The clinical data model consisting of demographics (age, sex, and education), MMSE score, ADAS delayed word recall and *APOE* status had a mean test AUC of 0.866 (CI 0.777–0.942) for predicting four-year progression to AD dementia. Adding intracranial and hippocampal volume to the clinical data model increased the test AUC to 0.910 (CI 0.828–0.975). When instead adding the FreeSurfer brain region volumes to the clinical data model, the test AUC increased to 0.902 (CI 0.826–0.962). Conversely, adding whole brain MRI and JD images to the clinical data together in a CNN model led to a slightly decreased test AUC of 0.862 (CI 0.772–0.938). However, when testing if the differences of the four main models’ performances on the test set are statistically significant, based on the ten models from the 10-fold cross-validation, it was found that both the hippocampal volume model and the FreeSurfer model were significantly better (p-value < 0.05) than the other two models, while there was no significant difference between any other pair of models. The results are presented fully in Supplementary Table 1 and visualized in Fig. 2 and Fig. 3.

### Sensitivity analysis of image modalities and multi-task learning included in deep learning model

The DL model described above included by default both the MRI image and the JD image derived from the image registration procedure. However, extracting JD images represents an extra, more burdensome processing step so we performed a sensitivity analysis in which the effect of fitting the DL model with MRI only or JD only. We found that using the JD image only, which had a mean test AUC of 0.605 (CI 0.418–0.766) for predicting four-year progression to AD dementia, outperformed a model using the MRI image only, which had a mean test AUC of 0.575 (CI 0.274–0.782). Moreover, we found that including both MRI and JD images had a mean test AUC of 0.609 (CI 0.366–0.766), thereby improving on the result from using the JD image only. The multi-task learning approach used was only evaluated for a limited number of *w*-values due to limited computational resources: *w* = **0,0.025,0.050,0.075,0.01**. The best value was determined based on the validation data, found to be *w*_1_ = **1**, *w*_2_ = *w*_3_ = **0.025**. All these results are displayed in Supplementary Table 1.

### Performance for predicting four-year decline in cognition

**Figure 4:**
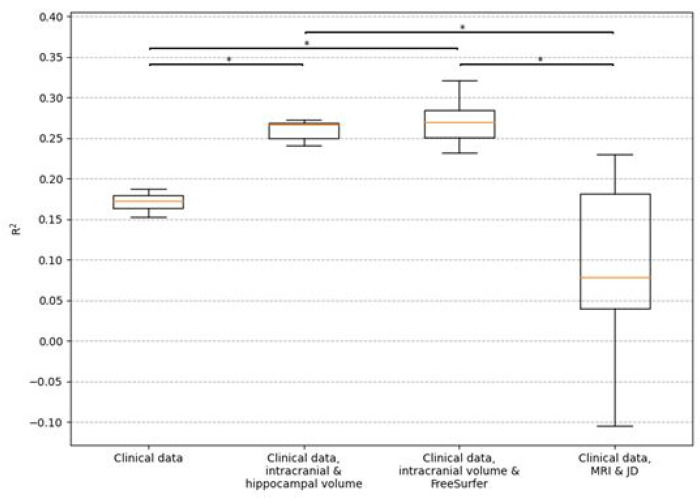
Box plots for R^2^ when predicting four years MMSE slopes. Results are for the test set using the different models from ten-fold cross-validation. The clinical data includes demographics (age, sex, and education), baseline cognition (MMSE score, ADAS delayed word recall) and *APOE* genotype. The orange lines show the median, the boxes span from the first quartile to the third quartile, and the whiskers extend from the box by 1.5 times the interquartile range. Significant differences (p-value < 0.05) are indicated with *.

**Figure 5:**
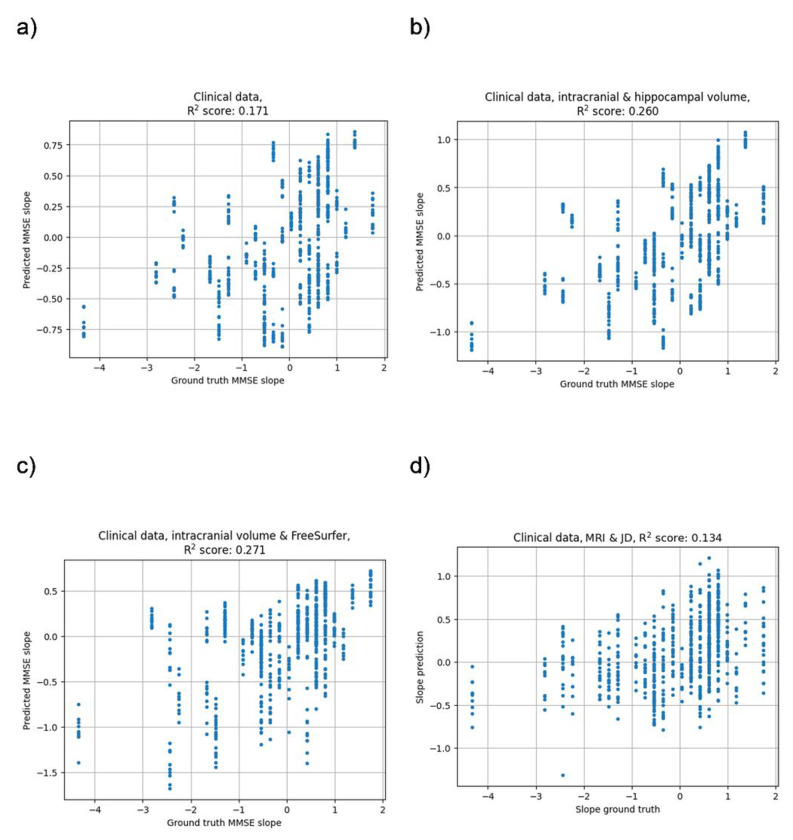
Correlation between predicted slope and corresponding ground truth. Models using a) clinical data, b) clinical data, intracranial volume and hippocampal volume, c) clinical data, intracranial volume and FreeSurfer, d) clinical data, MRI and JD.

The clinical data model consisting of demographics (age, sex, and education), baseline cognition (MMSE score, ADAS delayed word recall) and *APOE* status had a mean test R^2^ of 0.171 (CI 0.031–0.260) for predicting four-year cognitive decline as measured by MMSE. Adding hippocampal volume to the clinical data model improved the mean test R^2^ to 0.260 (CI 0.099–0.366). Adding FreeSurfer brain regions to the clinical data model improved the mean test R^2^ to 0.271 (CI 0.088–0.405). The DL model featuring the clinical data features and the whole brain MRI and JD images had the lowest mean test R^2^ of 0.134 (CI −0.023–0.256). The values for *w* that were found optimal for the previous task was used here as well but altered to prioritize this task (*w*_2_ = **1**, *w*_1_ = *w*_3_ = **0.025**). Just as for the models for predicting four-year progression to AD dementia, the hippocampal volume model and the FreeSurfer model were significantly better (p-value < 0.05) than the other two models for prediction of four-year decline in cognition, while there was no significant difference between any other pair of models. The results are presented fully in Supplementary Table 2 and visualized in Fig. 4 and Fig. 5.

### Identifying atrophy patterns from FreeSurfer and deep learning models

**Figure 6:**
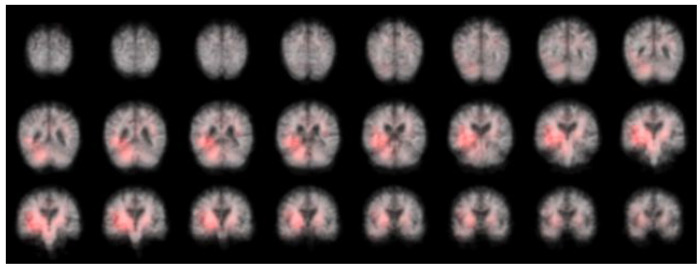
Heatmap with features used by the CNN. This figure shows the results of an occlusion experiment. Areas of the brain which are colored red are those areas whose occlusion led to a drop in model performance compared to when no occlusion was used.

Atrophy patterns representing the important brain regions of interest were identified for the DL whole brain model using the patch occlusion method described in the section “Statistical analysis”. Regions in the temporal and parietal lobes were identified, see visualization in Fig. 6.

## Discussion

We developed and evaluated different models for identifying SCD/MCI individuals who are more likely to progress to AD dementia within four years, as well as models for prediction of change in MMSE over four years. The models were based on combinations of demographics, standard cognitive tests, hippocampal volume, volumetric data from FreeSurfer, MRI 3D images and JDs computed from MRI. We focused on realistically evaluating model performances by following a rigorous approach without leakage of information from training to test sets, and we also focused on unbiased comparisons between models of different complexity. In general, we found that a model with only demographics and baseline clinical data (cognitive tests plus *APOE* ε4 status) performed very well, and that only smaller, although significant, improvements in predictive ability were seen when adding hippocampal volume or volumetrics data from FreeSurfer. We could not see consistent improvements in model performance when using the entire MR images in DL models. Taken together, this suggests that among the predictors tested here, most of the relevant predictive information for patients in the SCD/MCI stage of AD dementia is contained in the baseline cognitive profile together with an MRI assessment of hippocampal atrophy.

The problem of predicting progression to AD dementia has been studied multiple times before, both using MRI but also using data from e.g., PET. While PET has been shown to provide more information than MRI (19), it also has drawbacks such as being expensive and less available wherefore it was not used in this study. MRI in contrast is more readily available and a realistic option for prediction models in clinical practice. Previous publications have reported results with AUC values in the range 0.53–0.98 (19). However, it is hard to compare the studies in a fair way due to variations in for example imaging modality (e.g., MRI or PET), prediction task (e.g., MCI versus AD, or progressive MCI versus stable MCI with different follow-up times), the dataset used as well as the dataset’s division into development and test data. Our best-performing model with an AUC of 0.91 is within the range of previous reported results. Many studies are using data from the publicly available Alzheimer’s Disease Neuroimaging Initiative (ADNI) cohort (34), but due to different exclusion criteria and validation methods the final ADNI datasets vary. Furthermore, according to the review in (12), comparing studies performing classification of AD dementia using DL and MRI, it was found that there may have been data leakage and thus a bias in the reported results in more than half of the surveyed papers. It was shown that when correctly dividing the data such that data from the same subject was never present in both training and test set simultaneously, the accuracy dropped from 99–90% (10). We used data from the Swedish BioFINDER study, which has the major benefit of being a well-characterized cohort in terms of clinical diagnosis and biomarker confirmation of amyloid pathology as well as being more representative to a general memory clinic setting compared to the ADNI cohort (which is a more selected cohort, characterized for example by highly educated study participants, and few vascular co-pathologies). The test set was set aside from start and only used for evaluation after all models were finalized, thus not in any way influencing the development of the algorithm or model selection.

Prediction of MMSE slope is a less explored task than prediction of AD dementia. Compared to the binary classification task of separating non-AD and early AD, it has the advantage of being a continuous measure which is potentially less prone to bias and subjectivity compared to a clinical diagnosis. Moreover, cognitive assessment is relevant because it is typically the primary endpoint for clinical trials of AD.

We investigated whether predefined volumetric variables, such as hippocampal volume and volumetric variables from FreeSurfer, or data-driven features from MRI using DL optimized for the given task, provided the most information for prediction of SCD/MCI-to-AD progression and MMSE slope. While the MRI ought to contain at least as much information as the volumetric variables, since the volumetric variables are determined from the MRI, we anyway obtain results showing that the hippocampal volume or FreeSurfer variables give the best prediction for SCD/MCI-to-AD progression as well as MMSE slope prediction. The reason for this is probably that the amount of data available for training is too small to learn more representative features using DL and that the model’s capacity is too limited. The limited capacity was chosen to avoid overfitting and define a model suitable to the amount of available data. The results show that more data is needed for DL models to be successful. Thus, the results should not be interpreted as a failure for DL, but rather that its success might require an order of magnitude more data. Furthermore, it has been established for a long time that pathological changes in AD are focused in the hippocampus (35).

The DL model used in this work is based on the network developed by (14), which uses parameter-efficient layers such as grouped and separable convolutions. However, a few modifications were done to optimize it for our settings. Similar to the original network we used multi-task learning, but for other tasks. Instead of simultaneously predict SCD/MCI-to-AD progression as well as classify AD dementia vs healthy controls using a dataset with normal, stable MCI and progressive MCI cases, we use a dataset with only SCD/MCI cases and predict SCD/MCI-to-AD progression together with four years MMSE slopes and hippocampal volume. We show that the multi-task learning approach improves the performance (Supplementary Table 1). Training the model for all tasks simultaneously has a regularizing effect on the training and reduces the risk of overfitting the model to the relatively small dataset. Furthermore, it is likely that different tasks find descriptive information in similar features, hence the multi-task learning should not limit the models’ capacities.

Our best-performing model for prediction of SCD/MCI-to-AD progression utilizes the clinical data together with the hippocampal volume. However, the clinical data model alone performs very well and, in most cases, outperforms the other models which only utilizes one type of data (hippocampal volume, FreeSurfer variables, MRI or JD), Supplementary Table 1–2. A similar result was obtained in the study by (14), where they obtained an AUC of 0.79 when using MRI only and an AUC of 0.88 when using clinical variables only, similarly to our clinical data model.

While the atrophy patterns identified as important by the DL model differ for all patients, we can observe that the regions often coincide with regions that have been previously associated with AD dementia such as temporal and parietal lobes, see Fig. 6. The finding that the regions identified as important varied across patients is inherent to DL models compared to statistical models such as logistic regression that instead identify a common pattern of atrophy in the entire study population. The individualized nature of atrophy patterns derived from DL is a strong benefit because it can allow for personalized explanations as to how an individual’s predicted risk score was derived.

The main limitation of our study is the size of the dataset used, although it is similar in size (N = 332) to what has been used in some previous studies [(11): N = 968, (14): N = 785, (15): N = 509, (16): N = 559] but of higher quality with fewer healthy controls and AD dementia and higher ratio of SCD/MCI patients [our: N = 332, (11): N = 413, (14): N = 409, (15): N = 210, (16): N = 216]. Another limitation is the selection of model architecture and hyperparameters, which can be further explored. We investigated different amount of multi-task learning (Supplementary Table 1) and different techniques for normalization of the input data, but further optimization could be done. However, the network architecture used has been optimized in previous studies using another dataset (13, 14), and by not optimizing it further we reduce the risk of overfitting it to our study. Finally, the evaluation is limited by the gold standard, which is determined by the cognition variables and thus is biased towards those. Thus, it is not surprising that these variables have high correlation with the outcomes we use. The clinical gold standard is used clinically today and there is no similar metric that is based on for example volumetric features. However, it could be that such a metric also has a high correlation with both patients’ symptoms as well as our MRI based models. Another aspect of our work is that we did not test for conversion to all-cause dementia. Our aim was to develop tools that specifically predicted development of dementia due to AD. Due to the recent breakthroughs in DMTs against AD (7, 8), it is of high importance to specifically predict future development of dementia due to AD rather than dementia due to other diagnoses, since only patients at risk for developing dementia due to AD should receive these new types of treatment.

## Conclusions

We developed and evaluated four different models on two different outcomes. The models perform similar, but the clinical data model using only demographics (gender, age, education), baseline cognition (MMSE score, ADAS delayed word recall) and *APOE* status performs well and only small improvements can be seen when adding hippocampal volume or regional MRI gray matter volumes extracted using FreeSurfer. For identification of patients with high risk of SCD/MCI-to-AD progression within four years we obtained an AUC of 0.906 and for four-years MMSE slope prediction we obtained an R^2^ score of 0.141. The result for SCD/MCI-to-AD progression is similar to previous studies, while to the best of our knowledge no data are currently available with respect to prediction of MMSE slope. The best DL models identified uses multi-task learning, by being trained to simultaneously predict both SCD/MCI-to-AD progression, four years MMSE slope as well as hippocampal volume. We confirmed that the areas found as interesting by the DL models are reasonable using an occlusion algorithm. The results are humbling with respect to what can be achieved by DL models. In the future, it may be tested if better performance can be achieved by increasing the training sample size, or by adding additional investigational modalities or MRI-sequences, or by fine-tuning the outcome measures to minimize noise and variability.

## Figures and Tables

**Table 1:** Demographic of patients included in the study.

	Training & validation cohort		Test cohort	
	Non-AD	Early AD	Non-AD	Early AD
No. of subjects	267 (80.4%)		65 (19.6%)	
	179 (67.0%)	88 (33.0%)	44 (67.7%)	21 (32.2%)
MCI	75 (41.9%)	61 (69.3%)	17 (38.6%)	17 (81.0%)
Female	82 (45.8%)	43 (48.9%)	24 (54.5%)	8 (38.1%)
Age (years)	70.2 ± 5.4	71.8 ± 4.8	70.5 ± 6.0	73.2 ± 3.8
Education (years)	12.0 ± 3.6	11.8 ± 3.6	11.8 ± 3.9	12.1 ± 3.2
Baseline MMSE score	28.2 ± 1.7	27.2 ± 1.7	28.3 ± 1.4	27.0 ± 1.9
*APOE ε4*	0.4 ± 0.6	1.0 ± 0.7	0.3 ± 0.5	1.0 ± 0.7
ADAS-cog delayed recall (no of errors)	4.1 ± 2.4	6.5 ± 2.0	4.3 ± 2.5	7.5 ± 2.2
Hippocampal volume (mm^3^)	3327.1 ± 489.6	2972.6 ± 400.8	3386.7 ± 434.5	2738.3 ± 417.6
Intracranial volume (cm^3^)	1139 ± 158	1116 ± 111	1127 ± 131	1107 ± 91

Values are n (%) or mean ± standard deviation. Cross-validation was used, wherefore the training and validation data is presented together.

## Data Availability

Anonymized data can be shared to qualified academic researchers after request for the purpose of replicating procedures and results presented in the study. Data transfer must be performed in agreement with EU legislation regarding general data protection regulation and decisions by the Ethical Review Board of Sweden and Region Skåne.

## Abbreviations

AD: Alzheimer’s disease
ADAS: Alzheimer’s Disease Assessment Scale
ADNI: Alzheimer’s Disease Neuroimaging Initiative
AI: artificial intelligence
AUC: area under the curve
CI: confidence intervals
CNN: convolutional neural network
DL: deep learning
DMT: disease-modifying treatments
JD: Jacobian determinant
MCI: mild cognitive impairment
MMSE: Mini Mental State Examination
MRI: magnetic resonance imaging
SCD: subjective cognitive decline
